# The Sydney West Knowledge Portal: Evaluating the Growth of a Knowledge Portal to Support Translational Research

**DOI:** 10.2196/jmir.5786

**Published:** 2016-06-29

**Authors:** Anna Janssen, Tracy Elizabeth Robinson, Pamela Provan, Tim Shaw

**Affiliations:** ^1^Research in Implementation Science and eHealthFaculty of Health SciencesThe University of SydneySydney UniversityAustralia; ^2^Faculty of HealthDiscipline of Nursing and MidwiferyUniversity of CanberraCanberraAustralia; ^3^Centre for Cancer ResearchThe Westmead Institute for Medical ResearchSydneyAustralia

**Keywords:** knowledge management, web-based collaborative networks, translational research, cancer, capacity building, enabling factors

## Abstract

**Background:**

The Sydney West Translational Cancer Research Centre is an organization funded to build capacity for translational research in cancer. Translational research is essential for ensuring the integration of best available evidence into practice and for improving patient outcomes. However, there is a low level of awareness regarding what it is and how to conduct it optimally. One solution to addressing this gap is the design and deployment of web-based knowledge portals to disseminate new knowledge and engage with and connect dispersed networks of researchers. A knowledge portal is an web-based platform for increasing knowledge dissemination and management in a specialized area.

**Objective:**

To measure the design and growth of an web-based knowledge portal for increasing individual awareness of translational research and to build organizational capacity for the delivery of translational research projects in cancer.

**Methods:**

An adaptive methodology was used to capture the design and growth of an web-based knowledge portal in cancer. This involved stakeholder consultations to inform initial design of the portal. Once the portal was live, site analytics were reviewed to evaluate member usage of the portal and to measure growth in membership.

**Results:**

Knowledge portal membership grew consistently for the first 18 months after deployment, before leveling out. Analysis of site metrics revealed members were most likely to visit portal pages with community-generated content, particularly pages with a focus on translational research. This was closely followed by pages that disseminated educational material about translational research.

**Conclusions:**

Preliminary data from this study suggest that knowledge portals may be beneficial tools for translating new evidence and fostering an environment of communication and collaboration.

## Introduction

The term translational research is widely used in the health sector. It describes a spectrum of research from translating basic sciences into new treatment options for patients to improving quality of care by changing patient and health professional behaviors [1].

In spite of the widespread use of the term translational research, there is a low level of awareness regarding what it is and how to conduct it. This knowledge gap compromises the ability of health professionals to design effective translational research studies. The important role of translational research for translating evidence into practice to improve quality of care for patients makes this gap a cause for concern [2].

To address this gap the Sydney West Translational Cancer Research Centre (SW-TCRC) built an web-based knowledge portal: The Sydney West Knowledge Portal (SWKP). The SW-TCRC is a network of cancer care professionals and researchers in Western Sydney, New South Wales, Australia. The portal is a resource to support growth of the SW-TCRC and connect members who are dispersed by both geography and discipline. Additionally, it aims to disseminate key information to members in order to raise awareness and understanding of translational research and reduce the knowledge gap.

The SWKP is underpinned by the Westfall model of translational research. This model identifies 3 phases of research translation: T1, T2, and T3. T1 describes the translation of research from the laboratory to humans, T2 describes the translation to patients, and T3 describes the translation to practice [3]. This model is widely cited in the literature [4,5] and is also endorsed by the Cancer Institute NSW. The development of the SWKP was also informed by literature on the development of health professional networks [6] and strategies for optimizing knowledge translation [7].

A knowledge portal is a platform that drives knowledge production, integration, and management [8]. A distinction is made between knowledge and information. Knowledge represents what an individual knows. Information is knowledge that organizations and individuals can utilize in a meaningful manner. Although knowledge portals originated in the information technology sphere, they have since been more broadly adopted. In the health sector, they have been used for disseminating knowledge about quality improvement [9] and for improving patient health literacy [10]. There is also growing research interest into their use of tools to disseminate knowledge about translational research [11,12]. Knowledge portals are able to disseminate knowledge to a dispersed member base. They also have potential for increasing awareness and understanding of best practice in research translation.

This short report describes the preliminary design and growth of the SWKP, including an evaluation methodology to measure its reach and impact.

## Methods

An adaptive methodology was used to capture the design, growth, and evaluation phases of the SWKP. Adaptive methods modify aspects of the research in response to data received during the study, which is important when evaluating in a multiphase intervention like the SWKP.

### The Development Phase

Stakeholder consultation was undertaken with key individuals in the SW-TCRC. This process identified goals and boundaries of the portal by identifying optimal membership and member needs. The process also helped to identify the organizational objectives for the portal.

Data from the consultation process were analyzed and used to inform a portal schema. This schema underpinned the design of the portal and was used as a benchmark to evaluate how the portal evolved during its growth phase.

### The Growth Phase

This phase began when the portal was made publicly accessible. Membership of the portal was free to all members of the SW-TCRC network. Additionally, exclusive research support opportunities were promoted through the SWKP to encourage individuals to sign up. Word-of-mouth referral from cancer researchers was the primary means of recruitment for the portal. An email promotion campaign was also used to engage leaders within Western Sydney to encourage them to refer members. Finally, a grassroots advertising campaign was used to promote the portal across 3 hospitals within Western Sydney and at conferences and other scientific meetings.

The number of portal access requests made through the web-based sign-up form was analyzed to assess membership growth. Data from the application form were also used to determine distribution of members across the translational pipeline and their research focus. This form also provided referral information for each new member, allowing the research team to identify the portaling the research team to identify were used to determine member behaviors around the site and to identify the resources that attracted most traffic.

## Results

### The Development Phase

The development phase took 12 months to complete. At the start of this phase, a 3-month stakeholder consultation was undertaken. This included meetings with the management of the SW-TCRC, representatives of the organization’s Education and Training Sub-Committee, and a range of potential members including administrators, medical oncologists, nurses, and cancer researchers.

Meeting minutes from consultation sessions were analyzed and used to develop a schema document. This document proposed designing the portal around 3 pillars: Information, Community, and Education. Information would cover content on events, funding, and news specific to the SW-TCRC. Community would incorporate resources to encourage research collaborations or to share personalized content such as individual research projects. Education would cover content developed to transfer knowledge on research translation to support growth of research in this area.

The schema also identified features to incorporate into the portal design during the development phase. This included an activities section, discussion capabilities for member-organization interaction and member-member interaction, member blogs, and site notifications and newsletters to keep members connected.

The portal was built using the Refinery content management system (CMS). The CMS was chosen because it is free to use and adapt, meaning it could be customized to suit the needs of the SWKP. Additionally, it had a minimalistic administration interface, which made managing site content easier for SW-TCRC staff.

### The Growth Phase

The SWKP launched in February 2013. In August 2013, it reached its target of 200 members and was continuing to grow. By the end of 2013, the number of portal members had grown to 382. However, in the second half of 2014 membership growth began to slow down, and by early 2015 membership appeared to have equalized at 399 members.

Once the SWKP launched, governance was provided by the SW-TCRC Education and Training Sub-Committee, who reviewed the growth of the portal quarterly. Development of content for the SWKP was undertaken by a small team of editors within the SW-TCRC, under the guidance of implementation science and translational research experts. Additionally, SWKP members were given opportunities to contribute to the portal. One example of this was the publication of member-generated conference reports. Between February 2013 and December 2014, a total of 17 conference reports were published.

Analysis of member referral data showed that 21% (n=86) of current members referred new members. Of these, 50% (42/86) referred 1 new member, 21% (18/86) referred 2 new members, 16% (14/86) referred 3 new members, and 14% (12/86) referred 4 or more. Of the top 14% of referrers, 75% (9/12) were in leadership positions: 44% (4/9) referrers were directors, 33% (3/9) were managers, and 22% (2/9) were department heads.

Website analytics covering the first year and a half after portal deployment showed there were 7808 page views across 1154 sessions. The average session duration was 11 minutes and 6 seconds and consisted of 6 page views across the site. The majority of visits, 95.9% (1107/1154), were by returning visitors.

The top 50 pages across the 3 categories Information, Community, and Education were also reviewed. This showed that Community pages were the most popular, making up 27 of the top 50 visited pages on the portal (54%). This was followed by Education, which represented 9 of the 50 most visited portal pages (18%), and Information, which made up 5 of the 50 most visited pages (10%). A remaining 9 pages were classified as “other” (18%) and included pages such as “About SW-TCRC.”

## Discussion

Developing and sustaining knowledge portals is a significant challenge for organizations [13]. However, results of this study show that health professionals are interested in participating in such web-based communities. This is consistent with existing literature on the role of online research networks in health care [10,14,15]. Additionally, this study emphasizes the importance of the development phase in ensuring relevance and sustainability of web-based knowledge portals. A year was dedicated to designing the SWKP, which included development of key documents to clarify the technological and social structure that underpinned the community.

This paper also shows the important role of community members in growing and sustaining an web-based portal. In this study, members acted as content developers and as community recruiters. This finding contributes to the current literature on how knowledge portals act as foundations from which other web-based communities such as communities of practice and online collaborative networks grow [15].

Finally, SWKP metrics show that community-focused content is more appealing to members than other content. It was more than twice as popular as Education content and 5 times more popular than content in the Information category. Encouraging collaborations between health professionals is known to be challenging, and there is a lack of research into building and sustaining tools to do so [16]. SWKP data suggest that knowledge portals may be beneficial tools for fostering an environment of communication and collaboration.

## Abbreviations

CMS: content management system
SW-TCRC: Sydney West Translational Cancer Research Centre
SWKP: Sydney West Knowledge Portal
